# Height of Right and Left Ethmoid Roofs: Aspects of Laterality in 644 Patients

**DOI:** 10.1155/2011/508907

**Published:** 2011-10-19

**Authors:** Michael Reiß, Gilfe Reiß

**Affiliations:** ^1^Department of Ear, Nose and Throat, Elbandklinikum Radebeul, Heinrich-Zille-Stra*β*e 13, 01445 Radebeul, Germany; ^2^Department of Neurosurgery, University of Dresden, Ln, Fetscher-Straße 74, 01307 Dresden, Germany

## Abstract

*Objective*. The goal of the study was to determine the asymmetric distribution of the height of the ethmoid roof (fovea ethmoidalis). *Method*. We retrospectively reviewed 644 coronal sinus computer tomography (CT) scans. The height of the ethmoid roof was examined for possible lateral differences between the right and left sides. *Results*. In 221 CT scans (31%), there was an asymmetry between the height of the fovea ethmoidalis on the right and left side. Of these 221, 160 (72.4%) were lower on the right side, whereas 61 (27.6%) were lower on the left. The height of the ethmoid roof of the remaining 433 patients (66%) was symmetric. There were statistically significantly more asymmetric cases in men than in women (38% versus 29%). *Conclusions*. The present paper underlines the asymmetry, variability of the ethmoid roof, and the possible practical implications arising from that fact. The asymmetry of the roof of one side presents an additional point of consideration for careful preoperative and perioperative review of paranasal sinus CT scans in patients undergoing endonasal sinus surgery.

## 1. Introduction

The anatomy of the paranasal sinuses is very complex and the sinuses show great variability. The ethmoidal sinus is the most complex and variable of all paranasal sinuses [1–3]. Anatomically, the ethmoid sinuses are at the center of the paranasal sinus complex and communicate with the other sinuses. 

A helpful diagnostic instrument of paranasal diseases is computer tomography (CT) scanning of the paranasal sinuses and the base of the skull on several planes, that is, coronal or axial layers [3–7].

There are also some aspects of laterality or asymmetries in the region of the paranasal sinuses. The asymmetry of the frontal and the sphenoid sinuses and their structures are well known [8, 9]. However, there are also asymmetries of the anterior skull base and in particular in the ethmoid roof. It is an important part of the skull base with respect to intracranial complications during endonasal sinus surgery [4, 6, 10].

The ethmoid roof is primarily formed by the fovea or fossa ethmoidalis, an extension of the orbital plate of the frontal bone [4, 6, 10]. It is a very dangerous area with respect to surgical dissection [3, 11].

**Figure 1:**
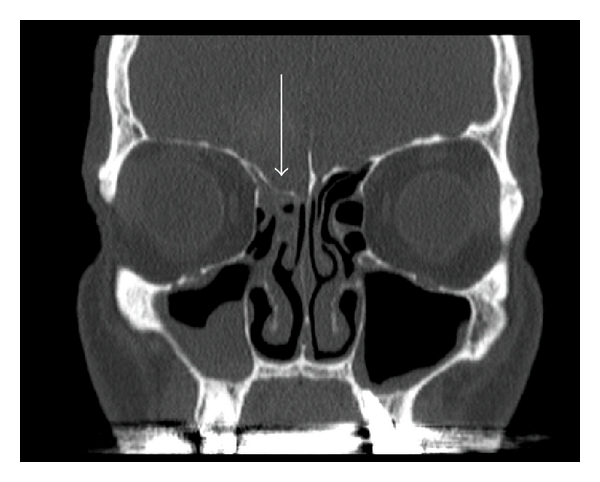
Ethmoid roof is lower on the right side (arrow). Coronary CT scan of a patient with chronic sinusitis.

The bone plates of the two sides may be at different levels (Figure 1). This may sometimes, but not always, be associated with a disease, for instance encephalocele. [4, 6, 10]. Differences in the depth of the fovea ethmoidalis were already shown in children aged up to 12 months [12].

**Figure 2:**
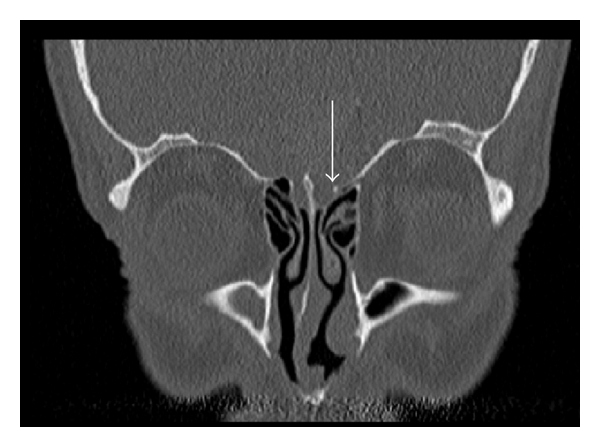
Ethmoid roof is “flattening” or shows a “broken wing” on the left side (arrow). Coronary CT scan of a patient with chronic sinusitis.

Contour differences of the fovea on one side are also possible. The contour is characterized by the degree of the angle at which the fovea ethmoidalis articulates on the cribriform plate. The fovea ethmoidalis has a “broken wing” or is “flattened” (Figure 2) when the angle is increased [6]. 

One important aspect for successful endonasal sinus surgery is the attention to details when dealing with the complex anatomy of the paranasal sinuses. Possible severe preoperative complications include injuries to the base of the skull or the orbita. Some studies have reported that complications or injuries occur more frequently on the right side of the base of the skull [11, 13, 14]. In particular, an asymmetric and lower lying fovea on the right side can be the cause of an injury of the anterior skull base. Such damage can cause a postoperative cerebrospinal fluid leak as well as meningitis [3, 11, 15, 16].

Up to now, published studies in the literature have investigated the asymmetry of the height of the ethmoid roof only in a relative small population (150 to 200 patients), and there is no information concerning gender differences [4, 6, 10]. Therefore, the goal of this study was to investigate the distribution of asymmetry of the height of the ethmoid roof or fovea ethmoidalis in a larger sample. An additional area of consideration is the investigation of possible gender factors.

## 2. Material and Method

The CT reports of 644 consecutive patients with sinus disease that were performed at several radiology centers and departments between January 2005 and December 2010 were reviewed retrospectively. Subjects were 381 male and 263 female patients with anamnestic and clinical signs of chronic sinusitis, ranging in age from 18 to 65 years old (mean = 39 years). All patients had endonasal endoscopic sinus surgery at the Department of Otorhinolaryngology of the Hospital in Radebeul, Germany.

Most CT images are available as films but not in digital format (90%). All CT exams were performed in the direct coronal projection. Images were inspected at bone window settings. We investigated all layers with specific attention to the height of the ethmoid roof. Three categories were distinguished (1) the height of the right and left roofs were symmetric, (2) the right roof was lower than the left one, and (3) the left roof was lower than the right one.

## 3. Results

160 (25%) patients had a lower ethmoid roof on the right side, 61 (9%) had a lower ethmoid roof on the left side, and 423 (66%) had a symmetric ethmoid roof (Table 1).

A lower ethmoid roof occurred more frequently on the right side in male patients (28% versus 20% for female) and a symmetric ethmoid roof was more prevalent in female than in male patients (71% versus 62% for male). The differences were statistically significant (*X* ^2^ = 6.17, *P* < 0.05, *FG* = 2).

When summarizing the cases with a lower ethmoid roof on the right and the left sides, there are 221 patients with an asymmetric roof and 423 cases with a symmetric roof. The differences between male and female patients are statistically significant (*X* ^2^ = 6.02, *P* < 0.025, *FG* = 1). Overall, women showed a more symmetric ethmoid roof than men.

## 4. Discussion

In addition to the clinical exam and nasal endoscopy, CT images are essential tools for the preoperative evaluation of the nose and the paranasal sinuses. The goal of the CT scan is to determine pathological changes as well as anatomic abnormalities. The CT scan documents the anatomical configuration for the benefit of the surgeon performing the endonasal sinus surgery. The fovea ethmoidalis is the upper limit of dissection in endoscopic surgery [3, 13, 16, 17].

Endonasal endoscopic or microscopic surgery of the paranasal sinuses has primarily been developed for the treatment of chronic sinusitis. Endonasal sinus surgery includes the use of technical improvements, such as laser, and is widely utilized for indications other than chronic sinusitis, that is, for the treatment of trauma or neoplasia [3, 15].

Computer tomography is not only an important diagnostic tool [3, 5, 11, 15], but is also a very simple method of investigating the asymmetrical height of the ethmoid roof [4, 6, 10].

The anterior cranial fossa or base of the skull can be perforated during endonasal surgery which may lead to brain damage, hemorrhage, and cerebrospinal fluid leakage during or after surgery. The fovea ethmoidalis and especially its height configuration is the most dangerous locus [3, 11, 15].

In the present study, 221 scans (31%) had asymmetry between the height of the ethmoid roof on the right and left sides. Of these 221, 160 (25%) were lower on the right side, whereas 61 (9%) were lower on the left. Of course, the differentiation into three main groups is relatively simplistic. As of recently, it has become possible to analyze the ethmoid roof using digital volume tomography [17]. But this was not the actual goal of the study. A further aspect or reason was that only 10% of the CT images were available in digital format.

In our sample, there were statistically significantly more asymmetric cases in males than in females (38% versus 29%). This difference may suggest, for instance, hormonal factors in the development of the craniofacial asymmetry.

In the literature, there are only a few comparable studies that investigate the asymmetry of the ethmoid roof. Lebowitz et al. [6] differentiated between the asymmetry in the height and contour of the ethmoid roof, that is, the angle between the lateral lamella of the ethmoid roof and the cribriform plate. The authors found a symmetry in 86 of 200 cases. 19 patients showed an asymmetry of the height of the ethmoid roof. 12 (63.2%) were lower on the right side, 7 (36.6%) were lower on the left side. 96 (48.0%) demonstrated a contour asymmetry with “flattening” of the ethmoid roof on one side (46 on the right and 50 on the left). One patient demonstrated both height and contour asymmetries. The study presents only a slight dominance of the right side with respect to height, but this was not the case regarding contour. Altogether, there were 58 patient with an asymmetry in the height or contour of the right side, and 57 with a symmetric distribution. This result suggests equal distribution of both sides and not a predominance of one side.

Dessi et al. [4] studied CT scans of 150 patient regarding the height of the ethmoid roof and they found an asymmetry in 15 patients (10.0%). 8 were male and seven were female. There were no further details regarding the distribution of gender. In 13 cases (8.6%), the right ethmoid roof was lower than the left, and in 2 cases (1.2%) the left roof was lower than the right. Thus, the authors found a dominance of the right side concerning the height of the ethmoid roof.

Kizilkaya et al. [10] studied the relationship between handedness and height of the ethmoid roof. They found that in 43 cases (25.90%), the ethmoid roof was lower on the right side, in 20 (12.05) lower on left side, and in 103 cases (62.05%), it was symmetric. Of 128 right-handed subjects, 41 (32.03%) had the ethmoid roof lower on the right side, 6 (4.69%) had the ethmoid roof lower on the left side, and 81 (63.28%) had a symmetric ethmoid roof. Of 17 left-handed subjects, 2 (11.76%) had the ethmoid roof lower on the right side, 14 (82.69%) had the ethmoid roof lower on the left side, and one left hander had a symmetric ethmoid roof. All 21 ambidextrous had a symmetric roof. There were no asymmetric ethmoid roofs in ambidextrous. In conclusion, Kizilkaya et al. [10] suggest that a relationship exists between handedness and height. However, the sample of left handers containing 17 people was relatively small.

In contrast to the study by Lebowitz et al. [6], the studies by Dessi et al. [4] and Kizilkaya et al. [10] do not consider the asymmetry of the contour. We have observed in our patients that an asymmetry of the contour is often associated with an asymmetry of the height of the ethmoid fovea. The transitions between height and contour asymmetry of the ethmoid fossa are fluid, therefore, we did not differentiate these two types of asymmetries.

The present investigation confirms the results of the two studies by Lebowitz et al. [6] and Kizilkaya et al. [10] suggesting a relevant asymmetry of the ethmoid roof. Overall, right-left differences in the height of the roof are a simple tool for documenting skull asymmetries or specifically the asymmetries of the base of the skull.

An injury of the base of the skull with cerebrospinal fluid leaks occur more frequently when an endonasal sinus surgery is being performed on the right side [4, 11, 13]. Fortunately, no injury or cerebrospinal fluid fistula has appeared in our 644 patients because all surgeries were performed by experienced specialists. Furthermore, the type of handedness can play a special role in surgery, particularly in the training phase [18]. We found no systematic studies in the literature which examined the role of handedness in this area. However, we think that not the handedness is so important but rather which side of the patient the surgeon is standing during surgery. Performing surgery on the left side of the paranasal sinuses is easier for the surgeon than performing it on the right side, if he/she is standing on the right side of the patient. It is better to start the surgery on the more difficult right side, when the surgeon is more focused and attentive [3, 4, 11]. The fact that in 8 to 26 percent of the cases, the ethmoid roof is lower on the right side than on the left side might furthermore explain why the incidence of the respective complication is higher on the right side. In addition, a possible lower or flattened and particularly dehiscent ethmoid roof on the right side is another reason to prepare for surgery on the right side with special care. The possibility of intracranial penetration on the side with a very low fovea during endonasal sinus surgery is much higher. The asymmetry of the height and also the contour of the ethmoid roof should always be taken into account preoperatively by careful review of the CT scans of the paranasal sinuses.

## 5. Conclusions

The present paper underlines the asymmetry, variability of the ethmoid roof, and its possible practical implications. We can confirm a relevant asymmetry of the ethmoid roof. According to the literature, the ethmoid roof overall is lower on the right side as compared to the left side in 8 to 26% of the cases.

Furthermore, there were significantly more asymmetric cases in men than in women in our sample (38% versus 29%). A possible asymmetrical ethmoid roof should be considered during all sinus surgeries.

## Figures and Tables

**Table 1:** Distribution of the height of the ethmoid roof with respect to asymmetry and symmetry in the sample (relative values in parentheses).

	Male	Female	Total (male and female)
Lower right ethmoid roof	107 (.28)	53 (.2)	160 (.25)
Lower left ethmoid roof	38 (.1)	23 (.09)	61 (.09)

Total asymmetry (lower right or left ethmoid roof)	145 (.38)	76 (.29)	221 (.31)

Symmetry	236 (.62)	187 (.71)	423 (.66)

Total (asymmetry and symmetry)	381	263	644
